# Amino Acids As Mediators of Metabolic Cross Talk between Host and Pathogen

**DOI:** 10.3389/fimmu.2018.00319

**Published:** 2018-02-27

**Authors:** Wenkai Ren, Ranjith Rajendran, Yuanyuan Zhao, Bie Tan, Guoyao Wu, Fuller W. Bazer, Guoqiang Zhu, Yuanyi Peng, Xiaoshan Huang, Jinping Deng, Yulong Yin

**Affiliations:** ^1^Guangdong Provincial Key Laboratory of Animal Nutrition Control, Institute of Subtropical Animal Nutrition and Feed, College of Animal Science, South China Agricultural University, Guangzhou, China; ^2^Jiangsu Co-Innovation Center for Important Animal Infectious Diseases and Zoonoses, Joint International Research Laboratory of Agriculture and Agri-Product Safety of Ministry of Education of China, College of Veterinary Medicine, Yangzhou University, Yangzhou, China; ^3^School of Medicine, College of Medical, Veterinary and Life Sciences (MVLS), University of Glasgow, Glasgow, United Kingdom; ^4^Laboratory of Animal Nutrition and Health and Key Laboratory of Agro-Ecology, Institute of Subtropical Agriculture, The Chinese Academy of Sciences, Changsha, China; ^5^Department of Animal Science, Texas A&M University, TAMU, College Station, TX, United States; ^6^Chongqing Key Laboratory of Forage & Herbivorce, College of Animal Science and Technology, Southwest University, Chongqing, China; ^7^Changsha Medical University, Changsha, China

**Keywords:** amino acids, arginine, asparagine, metabolism, infection

## Abstract

The interaction between host and pathogen decidedly shapes the outcome of an infection, thus understanding this interaction is critical to the treatment of a pathogen-induced infection. Although research in this area of cell biology has yielded surprising findings regarding interactions between host and pathogen, understanding of the metabolic cross talk between host and pathogen is limited. At the site of infection, host and pathogen share similar or identical nutritional substrates and generate common metabolic products, thus metabolic cross talk between host and pathogen could profoundly affect the pathogenesis of an infection. In this review, we present results of a recent discovery of a metabolic interaction between host and pathogen from an amino acid (AA) metabolism-centric point of view. The host depends on AA metabolism to support defensive responses against pathogens, while the pathogens modulate AA metabolism for its own advantage. Some AA, such as arginine, asparagine, and tryptophan, are central points of competition between the host and pathogen. Thus, a better understanding of AA-mediated metabolic cross talk between host and pathogen will provide insight into fruitful therapeutic approaches to manipulate and prevent progression of an infection.

## Interaction Between Host and Pathogen

The interaction between host and pathogen has a profound effect on the outcome of an infection. The host senses the presence of the pathogen through recognition of pathogen-associated molecular patterns, which are highly conserved. Cells of the host recognize unique molecules present on pathogens *via* pathogen recognition receptors, including toll-like receptors (TLR), C-type lectin receptors, nucleotide-binding oligomerization domain-like receptors, retinoic acid-inducible gene-I-like receptors, and AIM2 (absent in melanoma 2)-like receptor (ALR) (1, 2). Pathogen recognition by immune cells of the host results in activation of a common set of cell signaling pathways, including nuclear factor-κB (NF-κB), activator protein-1, and mitogen-activated protein kinase (MAPK). These signaling pathways modulate the immune responses of the host against the pathogen that include production of proinflammatory cytokines/chemokines, migration of neutrophils, and secretion of antibodies (1–3). However, the pathogen is usually equipped to evoke countermeasures to inhibit the host’s immune responses. For example, pathogenic *Escherichia coli* inhibit the activation of NF-κB through its pathogenicity factors, such as NleH1 and NleB (4–6).

Knowledge about host–pathogen interactions is obviously critical for understanding the pathogenesis of infection; however, it generally overshadows knowledge of metabolic cross talk between host and pathogen. At the site of infection, which can be regarded as a closed system, host and pathogen share similar nutritional substrates and generate common metabolic products. The host depends upon nutritional substrates to support its immune responses against the pathogen, while the pathogen is also highly dependent on nutritional substrates for its physiology because most pathogens are unable to synthesize some nutritional substrates. For example, *Plasmodium falciparum* has completely lost its capacity for *de novo* biosynthesis of amino acids (AA), thus it depends primarily on AA scavenging from the host and through the catabolism of hemoglobin (7, 8). Usually, the host experiences significant metabolic alterations after the infection by a pathogen (9–11), and a slight change in metabolism at the site of infection will remarkably shape the outcome of an infection. For example, the host experiences a significant change in glucose metabolism to support immune responses against pathogens, such as activation of T cells and monocytes, activation of inflammasome signaling, and production of IL-1 β (12–14). The abundances of glucose and α-glucan in the host affect global gene expression of *Streptococcus suis*, including the virulence factor amylopullulanase, which promotes epithelial cell adherence (15). Fucose from the host’s intestinal microbiota affects the metabolism of enterohaemorrhagic *Escherichia coli* and its expression of virulence genes for intestinal colonization (16). In essence, the host modulates the availability of nutritional substrates or metabolic products to effect the progression of pathogen-induced infection, while the pathogen uses the same or similar substrates to sense the anatomical location and the physiological status of the host to adapt (17). For example, the host achieves a metal-limited environment during infection by expressing calprotectin which chelates manganese; however, *Acinetobacter baumannii* coordinates transcription of a manganese transporter to facilitate manganese accumulation and overcome the manganese limitation resulting from expression of calprotectin (18). Indeed, there is fierce competition for trace elements and metabolic precursors between pathogen and host. Therefore, the host experiences a significant alteration in metabolism during infection, including metabolism of glucose, fatty acid, and AA (19–21). Evidence for metabolic cross talk between a pathogen and its host was highlighted in a recent review by Olive and Sassetti (17); however, a number of key areas involving AA interactions between pathogen and its host require further in-depth research. In this review, we examine metabolic interactions between host and pathogen from an AA metabolism-centric point of view.

## AA Affects the Immune System of the Host

Numerous reviews indicate that AA metabolism shapes the host’s physiology, including growth, reproduction, and immunity. AA metabolism affects the physiology of the host by serving as an energy source for cells (e.g., lymphocytes, fibroblasts, and enterocytes), a basic substrate for protein synthesis, a substrate for production of regulatory molecules [e.g., nitric oxide (NO), polyamines, and creatine], a regulator for cell signaling pathways [e.g., mechanistic target of rapamycin complex 1 (mTORC1), MAPK, and NF-κB], and a regulator for host metabolism and intestinal microbiota (22, 23). Recent compelling results indicate that AA have a significant influence on immune responses of the host. For example, arginine or glutamine effect activation of the innate immune system, such as TLR signaling, secretory immunoglobulin A (SIgA), and Paneth antimicrobials, as well as activation of intestinal cell signaling pathways, such as NF-κB, MAPK, and PI3K–Akt (24–26). Indeed, glutamine promotes intestinal secretion of SIgA through the intestinal microbiota, and involving both T cell-dependent and T cell-independent pathways (27). These investigations revealed that AA influence the innate immunity of the host; however, the importance of AA in adaptive immunity during infection is not known. AA [e.g., leucine, glutamine, and gamma-aminobutyric acid (GABA)] are of critical importance in mediating T cell function, including activation and differentiation of T cells, especially for Th1 and Th17 cells (20, 28, 29). For example, extracellular serine is required for optimal T cell expansion even though the concentration of glucose is sufficient to support activation, bioenergetics, and effector functions of T cells (30). Mechanistically, the influence of AA on the immune system of the host may largely depend on mTORC1 signaling since AA-induced mTORC1 signaling is required for differentiation of Th17 cells and their expression of IL-17 (20). Collectively, available results indicate that AA are of critical importance for shaping the immune functions of the host, including innate immunity and adaptive immunity.

## AA Effect Growth and Expression of Virulence Factors by Pathogens

A pathogen requires AA to support its physiological functions, and alterations in AA availability have remarkable effects on growth of a pathogen and its expression of virulence factors. For example, the addition of asparagine to Dulbecco’s Modified Eagle Medium promotes activation of the streptococcal invasion locus (*sil*) in Group A *Streptococcus* (GAS) (31). Dietary content of glutamine significantly affects the burden of *Pasteurella multocida* and the expression of its virulence factors (32). A high content of glutamine increases the bacterial burden in all tissues analyzed, including the heart, liver, spleen, lung, and kidney (32). Glutamine promotes the expression of virulence factors in the lung, including ompA, pm0442, pm0979, plpE, and hasR, and expression of pm0442, plpE, and hasR in the spleen (32). Similarly, glutamine acts as an on/off switch for the induction of virulence genes of *Listeria monocytogenes* (33). There is no virulence gene transcription by *L. monocytogenes* when concentrations of glutamine in macrophages are below the threshold, while there is maximum transcription of virulence genes when concentrations of glutamine exceed threshold concentrations of glutamine (33). Inactivation of GlnPQ (a l-glutamine high affinity ABC transporter) results in complete arrest of glutamine uptake, a dramatic reduction in expression of virulence genes, and attenuated virulence in a mouse infection model (33). These results indicate the importance of AA, especially glutamine, in the growth of a pathogen and its expression of virulence factors. AA metabolism is also of critical importance for pathogens to overcome defensive responses of the host. For example, the host imposes manganese and zinc starvation during *Staphylococcus aureus* infection, impairing glycolysis in *S. aureus* because manganese and zinc are essential for the activitiy of certain glycolytic enzymes in *S. aureus* (34). Glucose and other sugars are the preferred carbon source utilized by *S. aureus* to generate energy, and impaired glycolysis decreases the growth of *S. aureus* (34, 35). However, *S. aureus* overcomes this deficiency by shifting away from metabolism of sugars as an energy source to the metabolism of AA for energy and to reduce demand for manganese and zinc (34). Collectively, AA significantly affect the growth of pathogens and their expression of virulence factors.

Assimilation of local nutrients is also essential for fungal pathogens to establish an infection in their mammalian host. AA metabolism is crucial to the pathogenicity of major fungal pathogens such as *Candida albicans*. Metabolic adaptation to the microenvironments of the host is associated with fungal morphogenesis, cell wall remodeling, biofilm formation and stress responses, commensalism, all of which influence progression of infections. Metabolic adaptation is regulated by complex transcriptional networks such as the general control of AA metabolism (GCN response) in fungal species such as *Saccharomyces* and *Candida* (36). The exposure of *C. albicans* to macrophages or neutrophils induces expression of a cluster of genes required for nutrient assimilation and AA metabolism, as well as genes associated with hyphal growth (ECE1), adhesion (HWP1), and adaptation to oxidative stress (SOD1 and CAT-1) (37–39). *Candida glabrata* also increases the biosynthesis of both arginine and lysine in response to their internalization by macrophages (40). AA catabolism stimulates hyphal morphogenesis in *C. albicans* (41). Moreover, our recent transcriptomic analyses revealed the critical role of metabolism of AA, such as arginine, proline, aspartate, and glutamate metabolism, on biofilm formation by *C. albicans* (42). The aspartate aminotransferase gene is a common member of these AA pathways and it is significantly upregulated in isolates have a high capacity for producing biofilm (42).

During host tissue damage and invasion, *C. albicans* secretes various aspartic proteases, which liberate AA from host proteins (43). Released AA form peptides that are then taken up by *C. albicans via* dedicated oligopeptide transporters (Opt1–8) and other membrane AA permeases (44). Moreover, during a glucose deficiency *C. albicans* exploits AA as a carbon source, excreting excess nitrogen in the form of ammonia, potentially altering the pH of the host environment, and thereby triggering hyphal development (41). This process can contribute to neutralization of the acidic environment in phagosomes of macrophages (45, 46). In *Cryptococcus neoformans* upregulation of expression of AA transporters is a survival mechanism within macrophages (47). Overall, in fungal species, there is tight coordination of nutrient sensing and metabolic pathways *via* transcriptional circuitry, which regulates the global activation of AA biosynthesis in response to the dynamic nature of local niches of infection.

## AA Metabolic Alteration After Infections

There are significant metabolic alterations in AA at the site of infection or even other anatomic sites during an infection. For example, a *C. neoformans* infection perturbs the content of cysteine in the human lung epithelial cell line (A549) after 6 h (48). *Plasmodium yoelii* infection alters concentrations of AA in plasma of infected mice, including increases in 10 AA (valine, leucine, tyrosine, phenylalanine, EOHNH2, histidine, proline, aspartate, glutamate, alanine) and decreases in five AA (citrulline, cysteine, methionine, 1-MeHis, and arginine) (49). *P. yoelii* infection also increases 21 AA in the liver of mice, including threonine, asparagine, and arginine (49). Similarly, *P. yoelii* infection affects the abundance of AA in red blood cells (8). Using NMR (nuclear magnetic resonance)-based metabolomics to study white spot syndrome virus infection in crayfish gills, the virus: (1) increases glutamate, alanine, and methionine at 1 h postinfection; (2) increases alanine, tryptophan, histidine, tyrosine, and methionine at 6 h postinfection; and (3) increases the abundances of alanine, valine, leucine, isoleucine, glutamate, glutamine, phenylalanine, tyrosine, threonine, and methionine at 6 h postinfection in crayfish hepatopancreas (50). Enterotoxigenic *Escherichia coli* (ETEC) infection reduces concentrations of isoleucine in the serum of piglets (9). In the jejunum, the abundances of six AA changes after an ETEC infection, which includes decreases in the abundances of glutamine, asparagine, citrulline, and ornithine, and increases in the abundances of glycine and GABA (51). Changes in concentrations of AA in serum of mice infected with porcine circovirus type 2 (PCV2) have been studied systematically using isotope dilution liquid chromatography (LC)-mass spectrometry methods (52). PCV2 infection increases concentrations of proline, ornithine, and methionine in serum on day 3 postinfection, while concentrations of aspartate, arginine, proline, lysine, valine, isoleucine, and leucine decreases on day 7 postinfection, and there is no effect of PCV2 infection on concentrations of AA in serum on either day 10 or 14 postinfection (52). Those results indicate significant changes in concentrations of AA in serum of the host, but the exact mechanism for the changes is not known. However, changes in concentrations of AA in serum after PCV2 infection may result from changes in either AA metabolism or AA transport. Among seven AA transporters (i.e., Slc6a14, Slc6a20, Slc7a5, Slc7a6, Slc7a7, Slc7a8, and Slc7a9) responsible for the transport of these altered AA, PCV2 infection decreases the expression of *Slc7a5* and *Slc7a6* in the jejunum on day 7 postinfection (52).

## AA as Mediators of Metabolic Cross Talk Between Host and Pathogen

**Figure 1:**
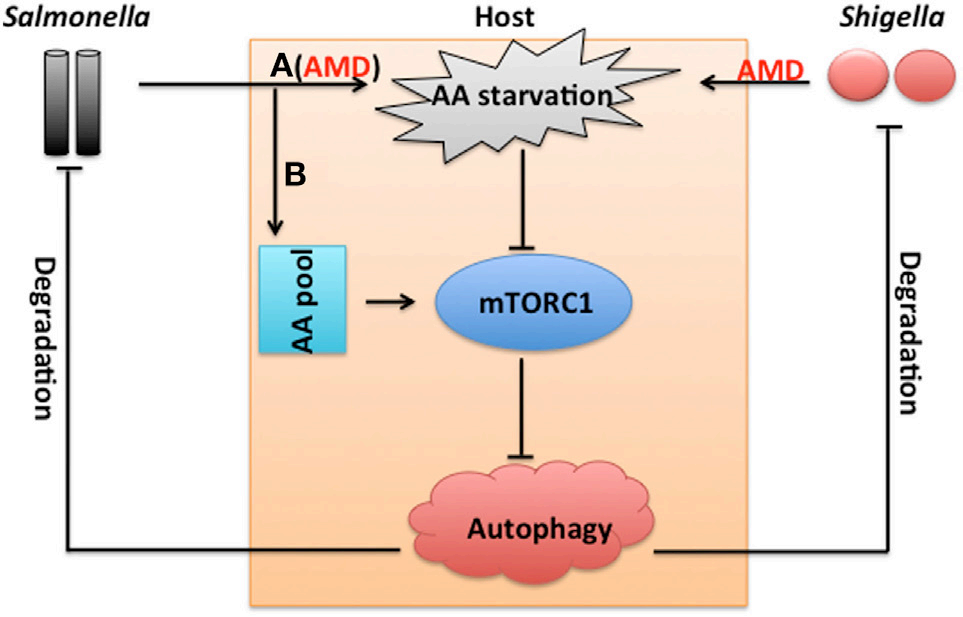
Extensive amino acid (AA) metabolism and communication between the host and *Salmonella* or *Shigella*. *Salmonella* (A, 1–2 h postinfection) or *Shigella* infection induces a rapid state of AA starvation through aseptic membrane damage (AMD), which inhibits the activation of mechanistic target of rapamycin complex 1 (mTORC1). mTORC1 negatively controls the autophagy response, which is responsible for targeting and degradating *Salmonella* or *Shigella*. However, *Salmonella* escapes from the autophagy-mediated degradation through replenishment of intracellular AA pools to reactivate mTORC1 signaling at a later phase of the infection (B).

A compelling example of extensive AA-dependent metabolic communication between host and pathogen occurs during infection with *Salmonella* or *Shigella* (Figure 1). Those infections rapidly induce a state of AA starvation in epithelial cells, which reduces abundances of leucine/isoleucine in cytosol, and inhibits activation of mTORC1, but induces the general control nonderepressible 2-dependent stress response pathway to promote expression of activating transcription factor 3 (ATF3) (53) (Figure 1). This starvation is induced through aseptic membrane damage, but not protein metabolism because chloramphenicol has little effect on the expression of ATF3 while inhibiting synthesis of bacterial proteins (53). AA starvation may inhibit the host’s innate immune responses and adaptive immune responses against pathogens, but activate host cell autophagy responses which are normally inhibited by mTORC1 (54) (Figure 1). Autophagy is a highly conserved cellular process that triggers nutrient recycling to sustain essential metabolic functions during nutrient or energy deprivation (55). Autophagy involves degradation and recycling of cellular constituents, such as dysfunctional organelles or macromolecular complexes (55). Autophagy also promotes targeting and degradation of intracellular bacteria (53, 56, 57), such as *Shigella* or *Salmonella*, and has important roles in the pathogenesis of extracellular bacterial infections, such as ETEC (58, 59). However, *Salmonella* could escape from autophagy-mediated degradation through the replenishment of intracellular AA pools to recruit and reactivate mTORC1 signaling at the surface of the *Salmonella*-containing vacuole (SCV) (53) (Figure 1).

A small change in cell and tissue content of AA after an infection has substantial effects on the ultimate outcome of the infection, and both host and pathogen can influence the availability of AA (e.g., asparagine, arginine, and tryptophan) and amounts of their metabolic products (e.g., NO, polyamines, kynurenine) at the site of infection to their respective advantages. The discussion will now focus on a few specific AA, arginine, asparagine, and tryptophan, which affect competition between host and pathogen, and strategies used by the pathogen to compete with the host for these AA, although the pathogen may also compete with the host for other AA.

### Arginine

**Figure 2:**
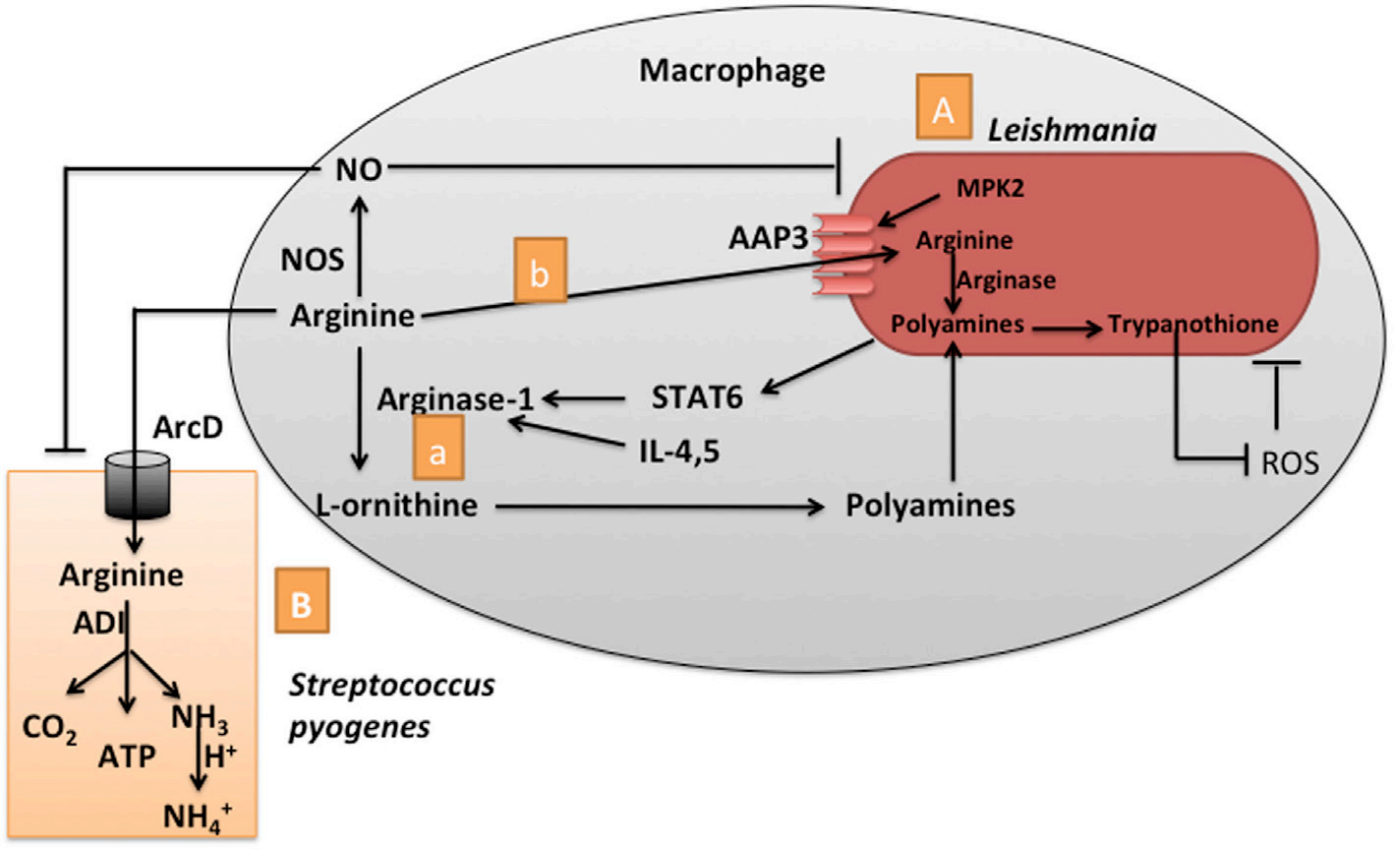
Competition between the host and *Leishmania* or *Streptococcus pyogenes* for arginine. **(A)** After *Leishmania* invasion, the infected macrophage produces nitric oxide (NO) from arginine *via* NO synthase (NOS) to kill *Leishmania* while *Leishmania* uses macrophage arginase I (a) to limit the amount of arginine available for synthesis of NO *via* NOS by macrophages. Mechanistically, the increase in Th2 cytokines during *Leishmania* infection, such as interleukin (IL)-4 and IL-5, induces the expression of arginase I in macrophages to produce polyamines. *Leishmania* directly activates signal transducer and activator of transcription-6 (STAT6) to increase the expression of arginase I in macrophages. *Leishmania* also uses the arginine transporter (amino acid permease 3, AAP3) (b) to compete with macrophages for arginine. During macrophage invasion by *Leishmania*, there is a coordinated arginine deprivation response mediated *via* the mitogen-activated protein kinase 2 (MPK2) cell signaling pathway, which upregulates arginine transport away from the macrophage. *Leishmania* uses polyamines to produce trypanothione that neutralizes effects of reactive oxygen species (ROS) released from macrophages. **(B)** *Streptococcus pyogenes* uses the arginine deiminase (ADI) pathway to limit available arginine for NO production by macrophages. This pathway includes the ArcD antiporter, which concomitantly transports ornithine out and arginine into the cell, enzymes which convert arginine to ornithine, CO_2_, NH_3_, and one molecule of adenosine triphosphate (ATP). The NH_3_ can be utilized to buffer against acid stress.

Utilization of arginine by both host and pathogen represents a metabolic bottleneck which is critical in determining the outcome of a pathogenic infection (17). The two predominant pathways for arginine metabolism are *via* NO synthase (NOS) for NO production and *via* arginase and ornithine decarboxylase for production of polyamines (putrescine, spermidine, and spermine). NO is an antimicrobial molecule, while polyamines are essential for the proliferation of pathogens (e.g., *Leishmania*). During infection, macrophages increase expression of inducible nitric oxide synthase (iNOS) to produce NO from arginine for antimicrobial purposes (60). However, the activity of iNOS in macrophages is highly dependent on the abundance of arginine in host cells (e.g., macrophages) and this influences the fate of an infection (61, 62). Arginase competes with iNOS for arginine; therefore, many pathogens exploit this to block NO production by increasing expression of arginase to limit arginine availability for metabolism *via* iNOS (61–63). One compelling example of the extensive competition for arginine between a host and a pathogen occurs during infections with *Leishmania* (Figure 2). Upon *Leishmania* invasion, infected macrophages activate cytotoxic pathways in an attempt to kill the pathogen, including the induction of NO biosynthesis from arginine; however, *Leishmania* infection upregulates arginase I activity in macrophages, thereby decreasing the availability of arginine for metabolism *via* iNOS (Figure 2) (64, 65). Mechanistically, the increase in Th2 cytokines during *Leishmania* infection, such as IL-4, IL-10, and transforming growth factor-beta, induces the expression of macrophage arginase I (64), or *Leishmania* directly activates signal transducer and activator of transcription-6 to promote expression of arginase I (66). Although pathogenic *Leishmania* (*L. major* and *L. tropica*) induces increases in arginase activity in susceptible BALB/c mice, and even in resistant C57BL/6 mice, there is no change in arginase activity at the site of infection and in the draining lymph nodes of either strain of mice during non-pathogenic *Leishmania* (*L. tarentolae*) infection (67).

Arginase in *Leishmania* can also subvert antimicrobial activity of macrophages by diverting arginine away from iNOS. *Leishmania* lacking arginase have poorer survival in mouse macrophages, and the decrease in intracellular survival is abrogated in iNOS-deficient macrophages (68). l-ornithine, the product of arginase, is used for the production of polyamines required for growth of *Leishmania* (64). In addition, *Leishmania* uses spermidine to produce trypanothione which neutralizes reactive oxygen species (ROS) released by macrophages (69) (Figure 2). Interestingly, an a comparison of arginase genes in pathogenic *Leishmania* (*L. major* and *L. tropica*) and non-pathogenic *Leishmania* (*L. tarentolae*) revealed that amino acid sequences of arginase from the pathogenic and non-pathogenic *Leishmania* are 98.6 and 88% identical to the reference gene in *L. major* Friedlin, respectively, and that the activity of arginase is greater in pathogenic than non-pathogenic *Leishmania* (67). *Leishmania, P. falciparum* infection also causes a rapid depletion of arginine through activation of arginase (8). Patients with *P. falciparum* infection have lower concentrations of l-arginine in their plasma and exhale less NO than controls, while l-arginine infusion increases concentrations of l-arginine in their plasma, exhaled NO, and other clinical indices without important side effects in *P. falciparum-*infected patients with malaria (70). Thus, a pathogen may affect the activity of arginase in the host and, therefore, arginine availability to cells of the infected host.

*Leishmania* also competes with macrophage for arginine by depleting arginine from phagolysosomes *via* changes in an arginine transporter (amino acid permease 3, AAP3) (Figure 2) (71). During macrophage invasion, there is a coordinated arginine deprivation response induced in *Leishmania* through a mitogen-activated protein kinase 2-mediated signaling pathway which upregulates expression of arginine transporters in *Leishmania* and arginine transport from the macrophage reduces available arginine for synthesis of NO *via* NOS by macrophages (71). *Salmonella* infection promotes the expression of arginine transporters in macrophages, including the cationic amino acid transporter members (CAT)-1 and 4 (61, 62). CAT-1 is preferentially localized in the cell membrane of uninfected macrophages, while CAT-1 is localized in close proximity to SCV in infected macrophages, which promotes the usage of arginine from macrophages by *Salmonella via* the arginine permease lysine–arginine–ornithine-binding periplasmic protein ArgT (61, 62). Interestingly, intracellular *Mycobacterium bovis* BCG also promotes the colocalization of CAT-1 to the intracellular BCG in macrophages (61, 62). Thus, *M. bovis* BCG may enhance expression of CAT-1 and CAT-2B for uptake of arginine into macrophages without a significant increase in NO production by infected macrophages (72, 73).

Pathogens also compete with macrophages for arginine *via* the arginine deiminase (ADI) pathway. The ADI pathway is critically important for *Streptococcus pyogenes* infection because *S. pyogenes* uses this pathway to increase the production of cellular energy and provide protection against acidic stress (Figure 2) (74, 75). This pathway includes the ArcD antiporter, which transports ornithine out and concomitantly brings arginine into the cell, and ArcA (ADI), ArcB (ornithine carbamoyl transferase), and ArcC (carbamate kinase) which convert arginine to ornithine, CO_2_, NH_3_, and one molecule of adenosine triphosphate (ATP) (75). NH_3_ can be utilized to buffer against acidic stress by reacting with protons from the fermentative metabolism of *S. pyogenes* and the accumulation of lactic acid. The ADI pathway mediates consumption of arginine and limits the availability of arginine for NO^⋅^ production by macrophages infected with *S. pyogenes* (Figure 2) (75).

Overall, during infection, the host uses arginine as a substrate to produce NO to protect itself against the pathogen, while, in parallel, the pathogen uses various mechanisms including arginase, arginine transport and the ADI pathway to deplete arginine and/or divert arginine away from host cells that produce NO (Table 1).

**Table 1:** Strategies used by pathogens to compete with the host for arginine, asparagine, and tryptophan.

AA	Strategies	Representative pathogen	Reference
Arginine	Arginase induction in host	*Leishmania*	(64, 65)
	Pathogen encoded arginase activity	*Leishmania*	(68)
		*Plasmodium falciparum*	(8)
	Upregulation of arginine and other transporters	*Leishmania*	(71)
		*Salmonella*	(61, 62)
	*Via* arginine deiminase pathway	*Streptococcus pyogenes*	(74, 75)

Asparagine	Asparagine synthetase induction in host	Group A *Streptococcus*	(31, 76, 77)
	Scavenge using asparagine transporter	*Mycobacterium tuberculosis*	(78)
	Pathogen encoded asparaginase activity	*Mycobacterium tuberculosis*	(78)
		*Salmonella typhimurium*	(79–81)

Tryptophan	Indoleamine 2,3-dioxygenase induction in host	*Clostridium difficile*	(82)
		*Mycobacterium avium* subsp. *paratuberculosis*	(83)

### Asparagine

**Figure 3:**
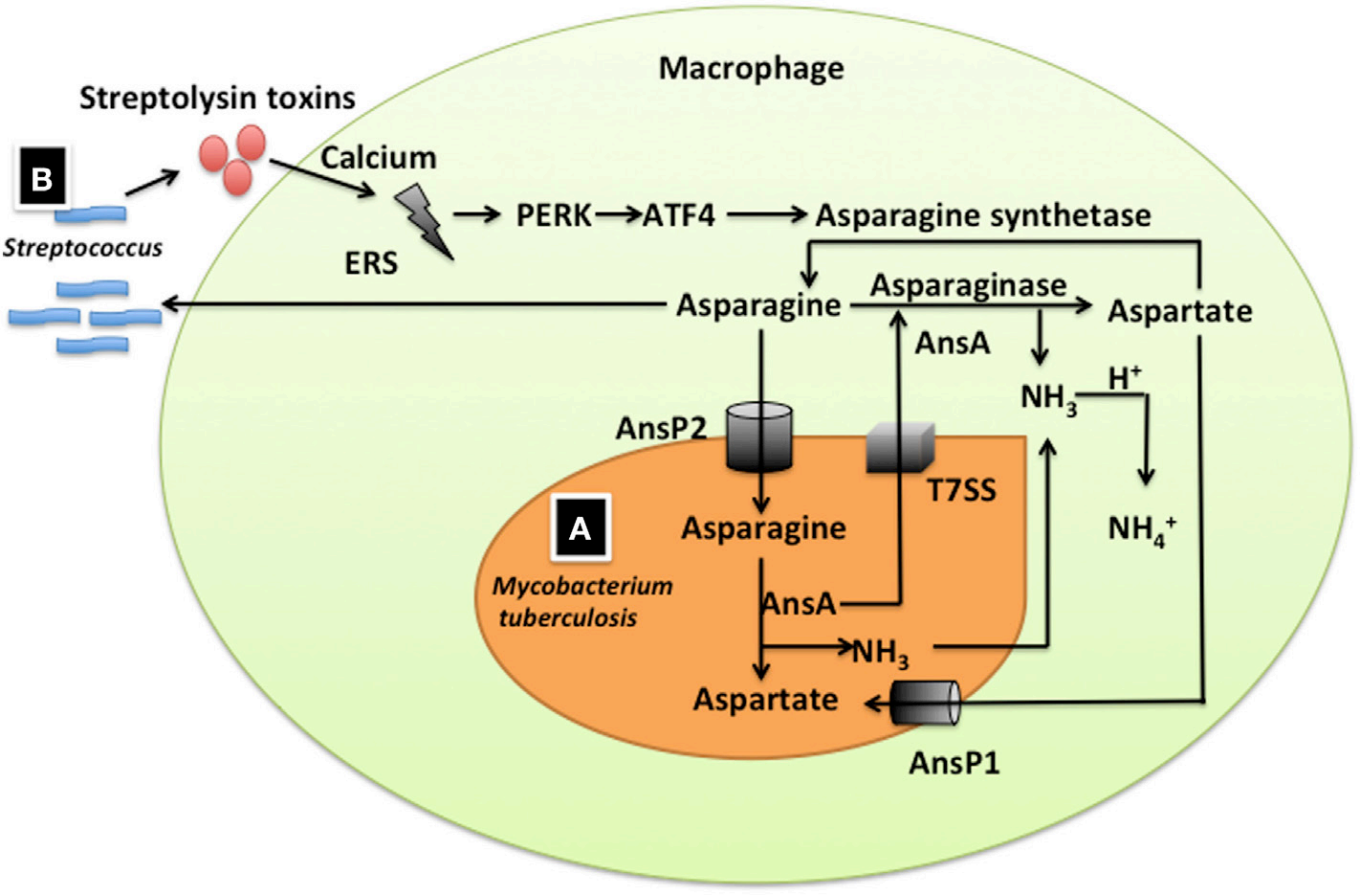
The role of asparagine in *Mycobacterium tuberculosis* and group A *Streptococcus* (GAS) infections. **(A)** Asparagine supports *M. tuberculosis* resistance to acid stress. *M. tuberculosis* expresses an asparagine transporter (AnsP2), which captures asparagine from macrophages, and asparaginase (AnsA), which hydrolyzes asparagine into aspartate and ammonia. Ammonia can react with protons in the phagosome to form ammonium ions. AnsA can also be secreted into the lumen of the phagosome of the macrophage through the type VII secretion system to hydrolyze asparagine to aspartate and ammonia. **(B)** Asparagine regulates GAS proliferation and expression of virulence factors. GAS infection creates endoplasmic reticulum stress (ERS) in host cells through effects of its streptolysin toxins on extracellular calcium signaling. ERS activates the protein kinase-like endoplasmic reticulum kinase (PERK), which induces the activation of the transcription factor activating transcription factor 4 (ATF4) to increase in the expression of asparagine synthetase and the production of asparagine. Asparagine promotes GAS proliferation and the expression of its virulence factors.

Asparagine is an important source of nitrogen for pathogens, especially GAS that is an extracellular pathogen infecting the human throat and skin. GAS creates endoplasmic reticulum stress (ERS) in host cells through its streptolysin toxins which causes transmembrane pores on host cells to permit extracellular calcium influx into the cytosol to dysregulate intracellular calcium (31, 84) (Figure 3). The ERS response activates the protein kinase-like endoplasmic reticulum kinase, which induces activation of transcription factor 4 and an increases expression of asparagine synthetase for production of asparagine (31, 76, 77) (Figure 3). Asparagine promotes GAS proliferation and changes the expression of about 17% of the GAS genes (including those for virulence, growth, and metabolism) partly through the two-component system TrxSR (31) (Figure 3). Asparaginase, which hydrolyzes asparagine into aspartate, blocks GAS growth in human blood and GAS proliferation in a mouse model of human bacteremia (31).

Asparagine is also essential for an intracellular pathogen (e.g., *Mycobacterium tuberculosis*) to resist acidic stress in a phagosome. Asparagine supports *M. tuberculosis* resistance to acidic stress by serving as substrate for production of the weak base ammonia, which reacts with protons in the phagosome to form ammonium ions (Figure 3) (78, 85, 86). *M. tuberculosis* expression of the asparagine transporter (AnsP2) increases markedly during infection to capture asparagine from macrophages (78). *M. tuberculosis* also expresses asparaginase (AnsA), which hydrolyzes asparagine into aspartate and ammonia (78). AnsA can also be secreted into the lumen of the phagosome of a macrophage through the type VII secretion system to hydrolyze asparagine for the production of aspartate and ammonia (78).

Besides its required role for pathogen survival and infection, asparagine is required for T cell activation. For example, the absence of asparagine in medium abolishes T cell activation induced by anti-CD3 and anti-CD28 antibodies, and also inhibits T cell blastogenesis and IL-2 secretion (80). Mechanistically, asparagine may affect T cell functions through mTORC1 which is critically important for T cell activation and differentiation, especially for Th1 and Th17 cells (9). Thus, the pathogen could inhibit the immune response of the host by inducing starvation of asparagine in host cells. For example, *Salmonella* Typhimurium infection induces asparagine deprivation by hydrolyzing asparagine *via S*. Typhimurium asparaginase, resulting in inhibition of mTOR signaling, Myc expression, T cell activation, and immune responses in the host (79–81). Using gas chromatography-mass spectrometry based metabolomics, ETEC was found to induce asparagine deprivation in the jejunum of piglets (51), but not in serum (9). This decrease may be responsible for inhibition of immune responses in the jejunum of piglets after ETEC infection (87). An ETEC infection inhibits activation of the NF-κB and MAPK pathways in the jejunum, and expression of TLR and other indicators associated with intestinal immunity, including phospholipase A2, lysozyme, polymeric immunoglobulin receptor, and mucin 2 (87, 88). However, in response to the ETEC infection, piglets try to alleviate asparagine deprivation by upregulating asparagine synthetase in the jejunum. Those results are based on the use of isobaric tags for relative and absolute quantitation (iTRAQ) combined with multi-dimensional LC and MS analysis (87). Interestingly, piglets with diarrhea have a lower content of asparagine in the jejunum than control piglets (51), whereas resistant and control piglets have similar contents of asparagine in the jejunum, and piglets that have recovered from ETEC-induced diarrhea restore their asparagine content, compared to piglets that did not recover from diarrhea (manuscript submitted).

Collectively, available results indicate that the competitive utilization of asparagine by pathogens *via* asparagine synthetase, asparagine transporter, and asparaginase (Table 1) has important influences on the pathogenesis of infection and the outcome of an infection.

### Tryptophan

**Figure 4:**
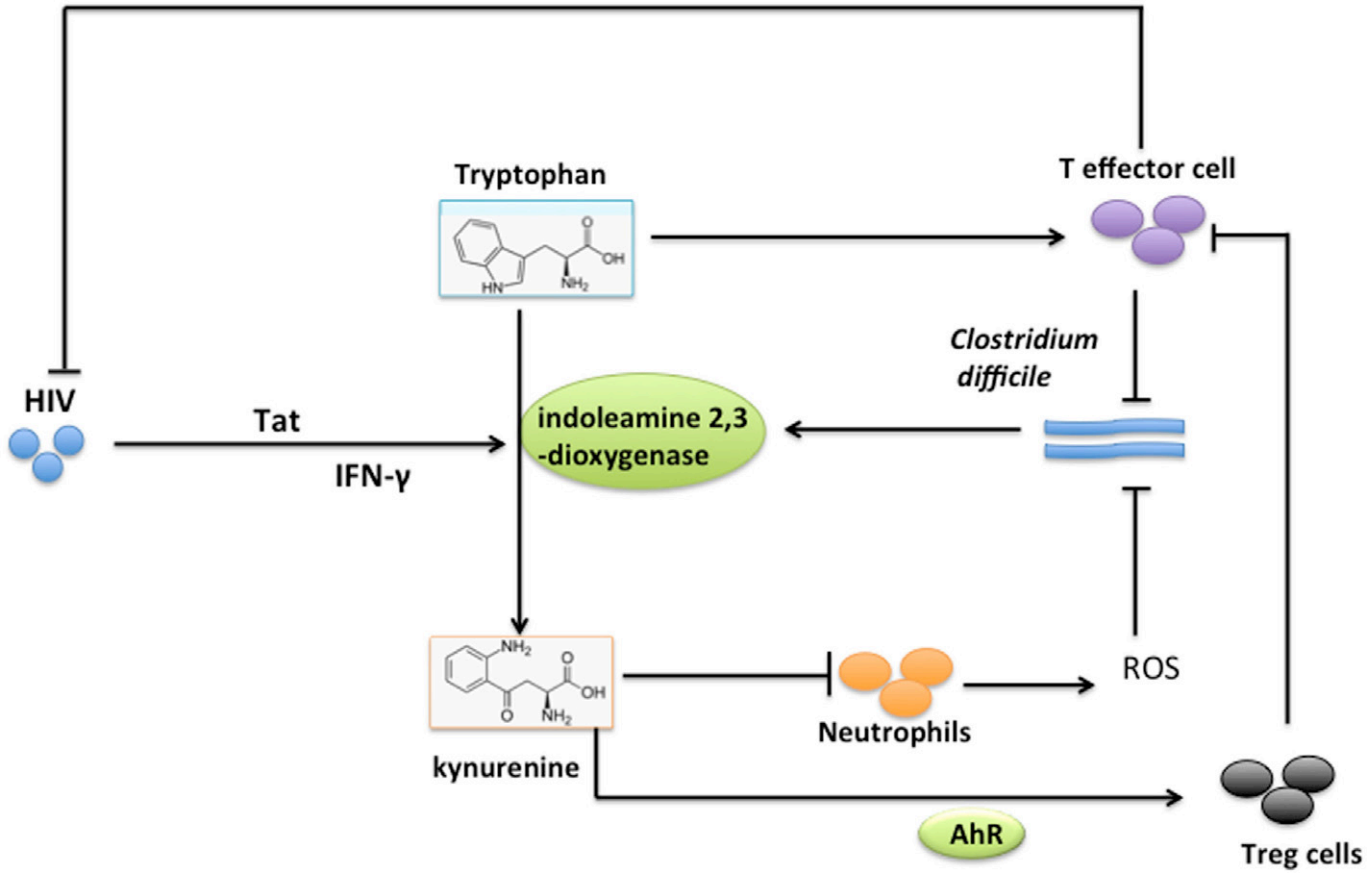
Tryptophan competition between host and *Clostridium difficile* or HIV. Upon *C. difficile* infection, tryptophan supports optimal effector T cell responses against *C. difficile. C. difficile* promotes activation of indoleamine 2,3-dioxygenase (IDO) *via* an unknown mechanism to convert tryptophan to kynurenine to deplete the tryptophan pool and diminish responses of effector T cells in the host. Kynurenine also promotes apoptosis of neutrophils, inhibits the production of reactive oxygen species from neutrophils, and enhances the generation of regulatory T cells (Treg) through the aryl hydrocarbon receptor (AhR). HIV induces expression of IDO through transactivator regulatory protein (Tat) and IFN-γ, leading to tryptophan catabolism, expansion of Tregs, loss of CD4^+^ T-helper cells, and functional impairment of T-cell responses.

Tryptophan is required for optimal immune responses, such as T cell proliferation (Figure 4). For example, activated T cells placed under tryptophan-deficient conditions undergo a mid-G1 arrest in cell cycle progression (89, 90). The tryptophan pool diminishes due to its conversion into kynurenine by indoleamine 2,3-dioxygenase (IDO) (Figure 4). Various regulators, including interferon (IFN)-α, IFN-γ, tumor necrosis factor-α, and prostaglandins, regulate the expression of IDO (91, 92). IDO has an immunoregulatory function in some situations, such as pregnancy, chronic infections, and tumors, by inhibiting T cell proliferation, increasing T cell apoptosis, and altering the balance of Th1 and Th2 cells (93, 94). The effect of IDO on immune responses may largely depend on availability of its substrate tryptophan and breakdown products of tryptophan (i.e., kynurenines). Kynurenine enhances the generation of regulatory T cells (Treg) through its interaction with aryl hydrocarbon receptors (95–97) (Figure 4). Kynurenine also promotes apoptosis of human and mouse neutrophils, and inhibits production of ROS (82) (Figure 4). Thus, a pathogen, such as *Clostridium difficile*, promotes activation of IDO to deplete the tryptophan pool of the host and diminish the immune responses of the host against the pathogen (Figure 4). A *C. difficile* infection induces the expression of IDO1 (4.7-fold) and production of kynurenine (about eightfold) in mouse intestinal lamina propria (82), while inhibition of tryptophan catabolism in IDO1-knockout mice increases the percentage of IFN-γ-expressing neutrophils and clearance of *C. difficile* from mice (82). *Mycobacterium avium* subsp. *paratuberculosis* infection promotes the expression of IDO in human monocytes in the gut and draining lymph nodes of sheep, and in peripheral blood cells of sheep and cattle, coincident with a decrease in amounts of tryptophan in those cells (83). HIV infection is associated with increased tryptophan catabolism (i.e., a high ratio of kynurenine to tryptophan in plasma and IDO1 activation), expansion of Tregs, and depletion of Tc17/mucosa-associated invariant T cells, while combination antiretroviral therapy in HIV-infected patients decreases tryptophan catabolism (98) (Figure 4). Further experiments demonstrated that HIV-induced IDO1 activation may be responsible for acute and progressive numeric loss of CD4^+^ T-helper cells and functional impairment of T-cell responses during an HIV infection (99). Mechanistically, the N-terminal domain of the HIV-1 transactivator regulatory protein (Tat) induces the initial expression of IDO, while increased IFN-γ during HIV-1 infection enhances the expression of IDO in monocyte-derived dendritic cells (DC) (100) (Figure 4).

Tryptophan is also essential for the growth of some pathogens, thus IDO has an antimicrobial activity by depleting cells of tryptophan. IDO is an anti-hepatitis B virus (HBV) effector based on evidence that patients with acute hepatitis B, but not hepatic flare, have robust activation of IDO (101). Further, patients with acute hepatitis who eventually clear HBV have increased IDO activity at the time of peak alanine aminotransferase activity, but patients with a hepatic flare-up have less IDO activity (101). Indeed, IDO promotes HBV suppression in Huh7 cells that is abrogated in IDO-knockout cells, but restored by re-induction of IDO in the cells (101). Inhibition of IDO in mice increases mortality and parasite burdens during *Toxoplasma gondii* infections (102). Depletion of tryptophan by high IDO activity causes *Chlamydia trachomatis* to lose infectivity and decrease transcriptional activity, but these outcomes are reversed in response to replenishment of cellular tryptophan pools (103). The suppression of IDO in the replication of intracellular pathogens also occurs in response to other pathogens, such as HIV (104), herpes simplex virus (105), and *Chlamydia pneumonia* (106). In parallel, some pathogens have evolved to synthesize tryptophan to protect them from local tryptophan depletion due to the increased expression of IDO during infection. *M. avium* subsp. *paratuberculosis* has the ability for tryptophan biosynthesis because it expresses genes for *trpA* (tryptophan synthase subunit alpha, MAP1307), *trpB* (tryptophan synthase subunit beta, MAP1306), *trpC* (indole-3-glycerol-phosphate synthase, MAP1305), *hisA* (phosphoribosyl isomerase A, MAP1297), *trpD* (anthranilate phosphoribosyltransferase, MAP1931c), and *trpE* (anthranilate synthase component I, MAP1303), which are all involved in the biosynthesis of l-tryptophan (83).

Collectively, tryptophan is required for both host and pathogen and IDO has biologically important roles in the interaction between host and pathogen, especially intracellular pathogens. IDO not only dampens protective mechanisms for host immunity to increase pathogen burdens but also suppresses replication of pathogens to limit the spread of infection. Thus, the dominant nature of IDO (i.e., antimicrobial or immunoregulatory) is pathogen-specific.

### Other AA

Our understanding of other AA involved in metabolic cross talk between host and pathogen lags far behind our understanding of the roles for arginine, asparagine, and tryptophan. There are sporadic reports on metabolism and regulation of other AA during infection. For example, human cytomegalovirus (HCMV) infection causes host cells to use glutamine to produce the tricarboxylic acid cycle intermediate-alpha ketoglutarate due to the removal of citrate from the citric acid cycle to increase fatty acid biosynthesis (107, 108). Thus, infection may induce changes in the energy substrate from glucose to glutamine, which is the major source of ATP in HCMV infected human fibroblasts, but not in uninfected cells (107). Glutamine deprivation inhibits the formation of infectious virions, while alpha ketoglutarate supplementation rescues ATP production and viral growth under conditions of glutamine deprivation (107). Also, glutamine uptake by the sodium-dependent neutral amino acid transporter 2 is required for motility and migration of *T. gondii* infected DCs (109).

Glutamate has a central role for bacteria due to its involvement in a wide range of metabolic processes. The glutamate-dependent acid resistance (GDAR) system is one of the best-characterized systems in commensal and pathogenic bacteria, including *E. coli, Shigella flexneri, L. monocytogenes*, and *Lactococcus lactis* (110). GDAR couples the glutamate decarboxylase(s) GadA and/or GadB, and glutamate:γ-aminobutyrate exchanger GadC (110). GadC has important roles for intracellular multiplication and virulence of *Francisella* because the Δ*gadC* mutant *F. novicida* exhibits impaired multiplication in macrophages, as well as in liver and spleen of mice (111). The Δ*gadC* mutant *F. novicida* is more sensitive to oxidative stress than the wild-type strain, but similar sensitivities to oxidative stress in a glutamate deficient medium (111). Further, Δ*gadC* mutant *F. novicida* loses the capacity to escape from the phagosomal compartment of infected macrophages due to a defect in its ability to neutralize ROS produced in the phagosomal compartment (111).

Thus, AA other than arginine, asparagine, and tryptophan can mediate interactions between a host and pathogen, and influence the outcome of the infection.

## Modulation of AA During an Infection

Amino acids are associated with interactions between a host and its pathogens and profoundly influence the outcome of infection. Therefore, AA therapy can be used to manipulate the progression of an infection. For example, a decrease in asparagine blocks GAS growth in human blood and GAS proliferation in a mouse model of human bacteremia (31). Glutamine deprivation inhibits formation of infectious virions of HCMV (107). In PCV2 infected mice with a reproductive defect, dietary supplementation with functional AA (e.g., arginine, glutamine, and proline) enhances host defense responses that reduce replication of PCV2 and improve reproductive performance (112–114). In *P. multocida*-infected animal models, dietary supplementation with arginine, glutamine, and proline promotes immune responses and decreases bacterial replication and their expression of virulence factors, as well as mortality (115–117). Collectively, AA influence the outcome of an infection by modulating the activation of host defensive responses and the growth of the pathogen or its expression of virulence factors. However, such knowledge should be transferred carefully in its application as a prophylactic measurement against pathogens. It is difficult to inhibit growth of a pathogen by deprivation of AA without inhibiting the immune responses of the host and it seems impossible to improve the immune responses of the host through replenishing of AA without promoting the growth of the pathogen. Thus whether adding or depleting one particular AA to favor the host to clear a pathogen depends on the specific metabolic situation associated with the pathogenic infection. Probiotics offer a promising surrogate to regulate the AA status of hosts. For example, the use of multi-species probiotics (*Bifidobacterium bifidum* W23, *Bifidobacterium lactis* W51, *Enterococcus faecium* W54, *Lactobacillus acidophilus* W22, *Lactobacillus brevis* W63, and *Lactococcus lactis* W58) can increase concentrations of tryptophan in serum and reduce the incidence of upper respiratory tract infections in individuals (118). *L. lactis*-derived GABA also modifies the expression of IL-17 in the intestine during ETEC infection (51). It would be of great interest to use genetically engineered probiotics to compete with pathogens in acquiring essential AA.

## Conclusion

There is an obligatory and extensive metabolic cross talk between host and pathogens. Upon infection, the host alters metabolism to support defensive responses against the pathogen, while the pathogen uses metabolic cues to sense its anatomical position and the immune status of the host (17). The host has the ability to alter AA metabolism after an infection by a pathogen (9, 52). Thus, AA influence immune responses of the host against a pathogen, such as the function of innate immune cells (e.g., macrophage), the activation and differentiation of T cells and the production of antibodies by B cells (27, 51, 112, 115, 116, 119). Also, AA play an important role in the physiology and virulence of pathogens. Thus, a change in AA metabolism at the site of infection will influence the outcome of an infection. Some AA, such as arginine, asparagine, and tryptophan, are central to competition between host and pathogen. However, the importance of other AA, such as glutamine, proline, and GABA, in the metabolic cross talk between a host and pathogen, must be explored further. For example, there is a significant change in GABA signaling during ETEC infection that affects the expression of IL-17 in the intestine during ETEC infection (51). Also, strategies and mechanisms used by pathogens to compete with the host for AA remain to be discovered (see Table 1). Understanding metabolic cross talk involving AA between host and pathogen will offer significant insight into pathogenic infections and reveal novel treatments to prevent and cure infections by modulating the abundance of AA and/or the metabolism of those AA.

## Author Contributions

WR, GZ, and JD conceived this study. WR, RR, and YZ wrote the manuscript. BT, YP, and XH provided critical discussion in manuscript preparation. FB, GW, and YY revised the manuscript.

## Conflict of Interest Statement

The authors declare that the research was conducted in the absence of any commercial or financial relationships that could be construed as a potential conflict of interest.

## Abbreviations

AA, amino acids; ADI, arginine deiminase; ADR, arginine deprivation response; AhR, aryl hydrocarbon receptor; ALR, AIM2 (absent in melanoma 2)-like receptor; AnsA, asparaginase; AnsP2, asparagine transporter; AP-1, activator protein-1; ATF, activating transcription factor; CAT, cationic amino acid transporter family; CLRs, C-type lectin receptors; DCs, dendritic cells; EHEC, enterohaemorrhagic *Escherichia coli*; ETEC, enterotoxigenic *Escherichia coli*; GABA, gamma-aminobutyric acid; GAS, group A *Streptococcus*; GCN2, general control nonderepressible 2; GDAR, glutamate-dependent acid resistance; HBV, hepatitis B virus; HCMV, human cytomegalovirus; IDO, indoleamine 2,3-dioxygenase; iNOS, inducible nitric oxide synthase; IL, interleukin; MAPK, mitogen-activated protein kinase; MPK2, mitogen-activated protein kinase 2; mTORC1, mechanistic target of rapamycin complex 1; NF-κB, nuclear factor-κB; NLRs, nucleotide-binding oligomerization domain-like receptors; NO, nitric oxide; NOS, NO synthase; PAMPs, pathogen-associated molecular patterns; PCV2, porcine circovirus type 2; PERK, protein kinase-like endoplasmic reticulum kinase; PRRs, pathogen recognition receptors; ROS, reactive oxygen species; RLRs, retinoic acid-inducible gene-I-like receptors; SCV, *Salmonella*-containing vacuole; SNAT2, sodium-dependent neutral amino acid transporter 2; STAT, signal transducer and activator of transcription; TLRs, toll-like receptors.
